# Selfhood and alterity: schizophrenic experience between Blankenburg and Tatossian

**DOI:** 10.3389/fpsyg.2023.1214474

**Published:** 2023-07-07

**Authors:** Alessandro Guardascione

**Affiliations:** School of Philosophy, University College of Dublin (UCD), Dublin, Ireland

**Keywords:** schizophrenic alienation, Blankenburg, Tatossian, intersubjective constitution, reflexivity

## Abstract

This paper presents a critical comparison between two phenomenological accounts of schizophrenic experience: on the one side, Blankenburg’s seminal work on the basal disturbance (*Grundstörung*) of schizophrenia as loss of natural self-evidence (*Natürlichen Selbstverständlichkeit*); on the other side, Tatossian’s insight, briefly elaborated in a lecture presented in Heidelberg in 1994 and largely forgotten by the relevant literature. Whereas the former mainly develops an *intersubjective* reading of schizophrenia, the latter suggests an *intrasubjective* understanding. Indeed, for Blankenburg, schizophrenic experience can be broadly characterized as a progressive impoverishment of our rootedness in the social world, leading to derealization and depersonalization. In this respect, Tatossian takes schizophrenic autism not as the effect of a loss of originary sociality but as the result of a deeper disproportion. For Tatossian, schizophrenia is characterized, ultimately, by a basic self-disorder or alteration that consists in the breakdown of the twofold dimension of transcendental subjectivity, encompassing both constituting consciousness and phenomenologizing onlooker. In this sense, his interpretation of schizophrenic disorders is closer to the ipseity-disturbance model. I show that while Blankenburg and Tatossian share a dialectical understanding of schizophrenia by pointing to basic modifications of the “transcendental organization” of experience, their divergence originates from a different reading of the phenomenological epoché. Except for the clinical perspective, the point of contention between Blankenburg and Tatossian seems to concern their use of internal resources of the Husserlian phenomenology. By presenting the philosophical presuppositions of their analyses, I discuss two key figures of phenomenological psychopathology by showing how their debate on the meaning of schizophrenic experience can be reframed by looking at the relationship between transcendental subjectivity and intersubjectivity in Husserl’s phenomenology.

## 1. Introduction

Since Blankenburg’s seminal work on the “basal disturbance”^1^ (*Grundstörung*) of schizophrenia as a loss of natural self-evidence (*natürlichen Selbstverstaendlichkeit*) (1971/2012), investigating the structural relationship between the Husserlian notion of phenomenological epoché and the schizophrenic alienation (*schizophrene Alienation*) has become a classical theme of phenomenological psychopathology (*cf.* Naudin et al., 1999; Sass, 2001; Rulf, 2003; Schwartz et al., 2005; Wulff, 2007, 2014; Brückner and Schlimme, 2014; Thoma, 2014). Yet, in a lecture presented in Heidelberg in 1994, Tatossian, among the leading figures of French psychopathology, sketches a different interpretation of the schizophrenic consciousness. Instead of focusing on the loosening of the *intersubjective* anchoring (*Verankerung*) into the lifeworld (*Lebenswelt*), like Blankenburg (1971/2012, V), Tatossian proposes an *intrasubjective* reading of schizophrenia. Since these authors share similar phenomenological perspectives on psychopathology (*cf.* Thoma, 2013), how is it possible to conceive two apparently opposite interpretations of schizophrenia?

In the first part of the paper, I discuss in what sense schizophrenia can be conceptualized as a disorder of intersubjectivity by briefly reconstructing Blankenburg’s account. I show why and how Blankenburg locates the basal disturbance of schizophrenia in the rootedness of the human being into the lifeworld. By explaining the role of intersubjectivity in the constitution of natural self-evidence, I then discuss why, for Blankenburg, there is a kind of formal isomorphism between the phenomenological epoché and the schizophrenic loss of common sense. In this sense, I show that Blankenburg draws on Husserl’s idea of transcendental intersubjectivity as the constitutional basis not just for any sense of objectivity or meaning-formation, but for the genetic conditions that make possible human experience. This is because transcendental intersubjectivity refers to the underlying processes and structures that enable shared meanings and norms, making communication and social interaction possible (*cf.* Zahavi, 1996, 1997, 2001; Duranti, 2010; Costello, 2012; Heinämaa, 2013; Szanto, 2016). In this respect, Blankenburg’s account of schizophrenia is based on the idea that disturbances of the syntheses of intersubjectivity affect the constitution of the social and cultural world and, consequently, the formation of personal identity (*cf.* Parnas and Handest, 2003; Stanghellini et al., 2017; Gilardi and Stanghellini, 2021).

In the second part of the paper, I present Tatossian’s alternative proposal, that, contrarily to Blankenburg and similarly to Kimura (1992), focuses on schizophrenia as a self-disorder, which entails an originary self-alienation and self-division (*cf.* Sass, 2001; Motobayashi et al., 2016). As I show, drawing mainly from Fink’s reading of the phenomenological method in Sixth Cartesian Meditation (1988/1995), Tatossian proposes to consider the schizophrenic alterations of experience, and in particular schizophrenic autism, as resulting from a split in the heart of transcendental life, which is characterized by the simultaneity of the *phenomenological onlooker* and the *constituting consciousness*. In particular, I rework Tatossian’s insights through an interpretation of the Husserlian notion of Ego-Splitting (*Ich-Spaltung*), here used to make sense of the model of schizophrenic experience proposed by the French psychopathologist.

It is worth stressing that while Blankenburg and Tatossian both adopt a transcendental approach to psychopathology, in the sense that they conceptualize schizophrenia by considering the conditions of possibility of anomalous and distressing experience (*cf.* Blankenburg, 1971/2012; Tatossian, 1979/2002; Legrand, 2019), they do not use pure phenomenological ideas. As for Minkowski (1933/1970), they do not simply transpose or apply philosophical ideas to understand individual cases (*cf.* Zahavi, 2021).^2^ Indeed, they do not use the epoché and the reduction to put aside theoretical psychopathological considerations. On the contrary, as I show, their use of phenomenological concepts and methods is strategic to make sense of patients’ lived experiences by drawing on clinical cases without disregarding empirical considerations. In particular, I claim that Blankenburg and Tatossian share a dialectical understanding of schizophrenia (Blankenburg, 1982, 2007; *cf.* Messas and Tamelini, 2018) by pointing to basal modifications of the “transcendental organization” (*transzendentale Organisation*) of experience.^3^ In this respect, their divergence originates from the attention to different constitutional poles of experience and from a diverse reading of the phenomenological epoché and reduction, which is discussed more as a performative act of the phenomenologist to draw analogies with schizophrenic consciousness rather than as a methodological presupposition of their analyses.

Therefore, in this paper, I show in what sense Tatossian’s account of the basal disturbance of schizophrenia simultaneously *contrasts and completes* Blankenburg’s approach. In particular, I claim that the point of contention between the two seems to concern the different use of the internal resources of Husserlian phenomenology. By presenting the philosophical presuppositions of their analyses, I discuss two key figures of phenomenological psychopathology by showing how their debate on the meaning of schizophrenic experience can be reframed by looking at the relationship between transcendental subjectivity and intersubjectivity in Husserl’s phenomenology. However, it is worth stressing that I am not interested in endorsing any of these two accounts. Indeed, I do not focus on the phenomenological problem of whether self-disturbances can be *de facto* conceived independently from impairments of the intersubjective syntheses. In this respect, as Sass, 2003, writes, “to distinguish with certainty between the core of an illness and its immediate sequelae (which may be compensatory or consequential) is an impossible task” (Sass, 2003, 169–170).

## 2. Blankenburg’s idea of schizophrenic alienation as a loss of common sense or natural self-evidence

Currently, there is a growing interest in psychopathology to understand how schizophrenia impacts the relationship between an individual’s sense of self, their relationships with other persons, and their engagement with the wider socio-cultural environment (*cf.* Fuchs, 2015; Fuchs and Röhricht, 2017). In this respect, Blankenburg was a pioneer in conceptualizing schizophrenia as a disorder of intersubjectivity (*cf.* Fuchs, 2014, 2015; Van Duppen, 2017; Thoma et al., 2022). Unlike most of the classical accounts centered on the idea of self-disturbances (*Ichstörungen*), Blankenburg did not view social cognition and reduced empathy as a result of a primary disturbance of experience that originates from within the individual (*cf.* Thoma and Fuchs, 2017, 2018; Thoma et al., 2022, 2). On the contrary, he conceived schizophrenia as a psychopathology of common sense by placing emphasis on impairments in the intersubjective dimension of experience (*cf.* Mishara, 2001; Summa, 2012, 2014).

Rather than focusing on hallucinations and delusions, Blankenburg was interested in persons affected by paucisymptomatic forms of schizophrenia, who provide consistent phenomenological descriptions of impairments of practical understanding (*cf.* 1969/2001, 305–307). In the early stages of schizophrenia, individuals frequently experience a tendency to question or challenge commonly accepted practices, customs, concepts, or ideas that are typically considered self-evident or taken for granted. Arguably, the shift of attention from positive to negative symptoms of schizophrenia allowed Blankenburg to reflect on insidious modifications of lived experience that were simply neglected or overlooked at the time (*cf.* Maggini and Dalle, 2018, 229–230). Already in an article from 1969, Blankenburg developed the idea that schizophrenic alienation can be conceived as a consequence of an atrophy or “abdication of common sense” (1969/2001, 305).

Far from simply designating a representational kind of knowledge, common sense plays a fundamental practical role in our everyday life, for it articulates our pre-reflective engagement with others and the world. Common sense is a background understanding rooted in the “habitual structure of the lived-body” (Fuchs, 2001, 323) that allows us to navigate social situations effectively, by recognizing when to speak and when to listen, interpreting nonverbal cues such as facial expressions and gestures, without needing to be self-aware of our own behavior (*cf.* Green et al., 2015). In this sense, common sense is crucial in everyday situations because it allows us to act appropriately in various contexts (*cf.* Thoma and Fuchs, 2017, 2018; Bizzari, 2018). Therefore, when common sense is disturbed or lost, individuals may struggle to understand and interpret social cues and expectations, and, in general, to orient even in simple social contexts (*cf.* Stanghellini and Ballerini, 2002). This can lead to a sense of dislocation and isolation from the social and cultural world (*cf.* Naudin et al., 1999; Mishara, 2001; Sass et al., 2018).

For Blankenburg, the progressive impoverishment of common sense found in persons affected by early forms of schizophrenia can lead to a general loss of “familiarity-with” (*vertraut sein mit*) the everyday world. This, in turn, generates a basic mistrust in reality (*cf.* Fuchs, 2014, 81; Binswanger, 1956). Actually, Blankenburg develops the idea that schizophrenia can be conceived as a loss of common sense or of the “self-evidence of the self-evident” (*die Selbstverständlichkeit des Selbstverständlichen*) through the case study of Anne Rau’s schizophrenia simplex (1971/2012, 89).^4^ Indeed, the very expression “natural self-evidence,” which gives the title to Blankenburg’s seminal monographic work, is derived directly from Anne’s own descriptions: “I simply find that I still need support. In all the simplest things of every-day life I need support. What I miss is natural self-evidence […] A lot of things are foreign to me. […] Every person is something. Everyone moves in a certain way. That is not the case with me. It’s simply about life, about leading a real life, so that one is not so outside”^5^ (1971/2012, 43, my translation).

Blankenburg uses different phenomenological concepts and methods to explore Anne Rau’s lived experience and existential condition. In particular, he distinguishes a descriptive, structural or eidetic, and constitutive or transcendental stage of phenomenological investigation (Blankenburg, 1980). Indeed, Blankenburg does not only rely on phenomenology to elicit first-person descriptions of Anne Rau’s lived experience by means of an empathic understanding meant to grasp what the patient feels or is aware of Jaspers (1913/1997, 55). Actually, he also seeks to uncover: (a) the essential and invariant features that characterize schizophrenia in general by adopting an eidetic approach meant to grasp “the (modifying) process, the processes which reveal the eidos of the abnormal one, i.e., the abnormal modification of being”^6^ (Blankenburg, 1980, 56); (b) the conditions of possibility of Anne’s way of being, searching for the genetic constitution of the schizophrenic disturbances by considering Anne Rau’s family and public life.

By drawing on personal memories and some interviews, Blankenburg observes that Anne’s experience is characterized by a persistent feeling of perplexity (*Ratlosigkeit*) (1971/2012, 81, 134). Indeed, Anne laments about having lost “something small” (*so etwas Kleines*) but essential for living (*Wichtigen*, *Grundsätzlichen*). She refers to those elementary “rules of the game” (*ibidem*, 102) commonly shared by others in social interactions, which constitute the “basic axioms of everyday life” (*cf.* Straus, 1958; Summa, 2012, 194). In particular, Anne laments for lacking a standpoint (*Standpunkt*), a support (*Halt*) for understanding other persons’ behavior and their life projects. Blankenburg’s exploration of schizophrenic alienation revolves around four interrelated transformations in Anne’s field of awareness: her experience of the everyday world, temporality, self-experience, and relationships with others. However, Blankenburg places particular emphasis on the intersubjective dimension of Anne’s interactions with others, whose analysis is the lengthiest of the four.

In this regard, Blankenburg observes that Anne finds it difficult to participate in the everyday life of mutual communication. In particular, Anne laments a loss of perspective-taking and position-taking, and a general emotional fragility. For Blankenburg, Anna has lost a fundamental reciprocity (*Gegenseitigkeit*) that naturally characterizes our daily encounters. Rather than experiencing a sense of belonging and connection, Anne is overwhelmed with a feeling of dismay or horror (*Entsetzen*) when engaging with others. This is evident in her struggle to stand their gaze, which makes her feeling disconnected from herself (1971/2012, 134). For Blankenburg, Anne’s general discomfort expresses a fundamental non-familiarity with the socio-cultural dimension and discloses the problematic character of her being-in-the-world. This type of non-familiarity and questionableness (*Fragwürdigkeit*) encompasses any relation she has with others (*ibidem*, 134–135). Blankenburg notes that Anne seems to be obsessed with the others’ sense of trust, confidence, and spontaneity because she cannot be a “world-forming force” (*weltbildende Kraft*) (*ibidem*, 138), namely, an active source of natural self-evidence.

In fact, for Blankenburg, Anne’s distress is not primarily the consequence of the lack of some specific knowledge or cognitive ability, as she demonstrates a constant tendency for self-reflection. Instead, Anna seems to have lost a kind of *naturality* of what we consider self-evident (*ibidem*, 80, 143). Indeed, if, on the one hand, Anna does not know how to live her life or to engage with others, on the other hand, for her it is almost indifferent to live one way or another (*ibidem*, 157). Broadly, self-evidence refers to things that are immediately and intuitively clear to us, without requiring further explanation or justification (*cf.* Audi, 2019). Self-evidence, or what we consider obvious and immediately understandable, is hidden in everyday trivialities, so the difficulty of its analysis concerns its elusive nature. Actually, as Blankenburg acknowledges, the relationship between evidence and non-evidence constantly changes in our life (Blankenburg, 1971/2012, 76–80). Indeed, what may seem obvious to us today may not seem so tomorrow.

When Blankenburg discusses natural self-evidence, he does not refer to a specific knowledge or understanding, which is determined by culture or societal norms. Rather, he considers natural self-evidence as a *basal quality* of our existence that constitutes the condition of possibility of an original sense of trust and confidence that we have in our ability to perceive, understand, and act in the world around us (*ibidem*, 83, 142;). Beyond our explicit awareness, natural self-evidence belongs to the pre-predicative and pre-reflective dimensions of our experience. Indeed, prior to any conscious reflection or linguistic articulation of our experiences, we have an intuitive and spontaneous way of apprehending and relating to the world that operates almost underneath our explicit awareness through our bodily existence (*cf.* De Haan and Fuchs, 2010; Maiese, 2016). In this regard, as I show in the next section, Blankenburg uses Husserl’s concept of the lifeworld and the phenomenological epoché to conceptualize and understand Anne’s distressing and anomalous experiences.

### 2.1. Framing natural self-evidence: the intersubjective dimension of the lifeworld and the phenomenological epoché

Husserl thematizes the lifeworld as the world of experience, which is “constantly valid for us, with unquestioned certainty, as simply there” (*vorhanden*) (1936/1970, 76). In particular, the lifeworld designates the intentional background that characterizes our natural attitude and constitutes “the ground of all our interests and life-projects” (*ibidem*, 154). In this sense, the lifeworld is also the world of our practical activities. Indeed, as Husserl writes, the lifeworld is the word “in which we live and in which we carry out activities of cognition and judgment, out of which everything which becomes the substrate of a possible judgment [that] affects us is always already pregiven to us as” (1939/1975, 42). Accordingly, Husserl designates the lifeworld as the “ultimate source of primal self-evidence” (Urevidenz) (1936/1970, 24, 123, 127) rightly because it is the source of any passive belief, including our habitual or taken-for-granted assumptions (1939/1975, §7). Husserl also uses the expression “protodoxa” (*Urdoxa*) to refer to the “non-modalized belief” of the “ultimate substrate” of pre-given objects of sensuous perception, constituting pre-cognition (*Vorbekanntheit*) and pre-knowledge (*Vorwissen*) (*cf.* Husserl, 1939/1975, §8, 387).

Yet, if Blankenburg draws on this concept, it is because the lifeworld defines the intersubjective background of any of our cognitive and practical activities, and, in this sense, it can designate “the intentional correlate of common sense” (Blankenburg, 1969/2001, 306). Indeed, Husserl does not conceptualize the lifeworld as the intentional background of isolated subjects, as if it were the individual correlate of one’s lived experience. Instead, while Husserl views the lifeworld as the world that shapes our unreflected understanding of reality, he also describes it as that world that is constituted through everyday interactions within the “community” of persons through a process of “communalization” (*Vergemeinschaftung*) of our experiences (1936/1970, §47, 161, 163). In this sense, Husserl takes the lifeworld as an *intersubjective accomplishment* because, as the background of everyday reality, it is the very same world of communication, education, tradition, and, in this sense, culture (1936/1970, 187, 1939/1975, 42).

Nevertheless, from the first-person perspective that characterizes the single individual, the lifeworld is the horizon within which we develop our practical knowledge and make sense of social norms, customs, and expectations. In this regard, it provides the ultimate ground of our social, cultural, and historical situatedness (*ibidem*, 371; Husserl, 2008, 496). As Husserl writes: “[…] history is from the start nothing other than the vital movement of the coexistence and the interweaving of original formations and sedimentations of meaning” (Husserl, 1936/1970, 371). Therefore, for Blankenburg, the loss of natural self-evidence observed in the early stages of schizophrenia is a loss of the pre-predicative world of experience that is intimately intersubjective since it constitutes the pre-knowledge of any intentional object that populate our common sense understanding of reality.

Moreover, our rootedness in the lifeworld does not only shape our knowledge or tacit understanding of reality, whether theoretical or pre-predicative. The intersubjective character of the lifeworld, as the world of culture and social interactions, also constitutes the condition of the possibility of our identity (*cf.* Husserl, 1952/1989; Čapek and Loidolt, 2021). Actually, for Husserl, the development of our identity presupposes a “communicative intertwinement” with others because it is only through social relations that we can confirm or correct our knowledge (Husserl, 1936/1970, 128) and, in particular, develop our commitments to determinate values and norms (Husserl, 1977, 164; *cf.* Zahavi, 2022, 276). As a matter of fact, our beliefs and values are not born *ex nihilo* from ourselves. Instead, they result from our confrontation with other peoples’ convictions and motivations within a determinate cultural context. Indeed, if we can feel part of a broader community of persons, it is because we can comprehend, adapt to, or reject the motivations and convictions of others without losing our sense of self (Husserl, 1977, 162–163).

Therefore, for Blankenburg, the weakening of our anchoring in the lifeworld as an intersubjectively constituted world of experience does not simply impact the common sense understanding of reality. It also disrupts the very development of personal identity, as exemplified by Anne Rau’s anxieties about developing a personal agency. Actually, whereas Blankenburg uses the notion of lifeworld to contextualize the role of natural self-evidence in our life, he uses the notion of phenomenological epoché to characterize the *loss* of natural self-evidence and, in particular, the experiential quality of schizophrenic existence. Blankenburg draws on this concept because, within phenomenology, the epoché constitutes the *dā ubi cōnsistam* for grasping natural self-evidence. Indeed, in *Ideas I*, Husserl introduces the *ἐποχή* as a “*methodic expedient*” modeled on the Cartesian doubt through which the phenomenologist can suspend the epistemic certainty of the world. In the monography, Blankenburg develops the idea of a kind of formal isomorphism between the schizophrenic alienation and the phenomenological epoché.

The phenomenological epoché consists of a “*radical alteration*” of our natural attitude, i.e., the ordinary way in which we take things for granted and accept the world as it presents itself to us without questioning its reality or validity (*cf.* Husserl, 1913/1983, 53). In the natural attitude, we hold the assumption that the world exists regardless of our perception, and we believe that our perceptions faithfully mirror the true nature of the world. Indeed, we do not usually question the reality of the objects and events around us or the validity of our perceptions. Instead, through the epoché, the phenomenologist suspends or “*puts out of action*” the commitment to the existence of a factual world, naturally at hand in his lived experience (*cf.* Husserl, 1913/1983, §31, 59).

By practicing the epoché, the phenomenologist brackets what he takes to be self-evident to investigate the actual content of what remains in the consciousness (*cf.* Luft, 1998; De Warren, 2020). Insofar as Husserl intends to return “Zu den Sachen selbst,” it is mainly an epistemological interest that guides the phenomenologist’s epoché (*cf.* Luft, 2004). The phenomenological suspension of all the unquestioned position-takings about the world is preparatory for an epistemic task, that of a presuppositionless investigation on the whole of experience. This investigation aims to uncover the essential features of our experience, and to reveal the underlying structures of consciousness (Husserl, 1913/1983).

Against this backdrop, Blankenburg develops the idea that schizophrenic alienation can be conceived as an extreme form of epoché. However, it is worth mentioning that the phenomenological and schizophrenic epoché have more differences than similarities. In his comparison, Blankenburg recognizes six differences between the phenomenological epoché and schizophrenic loss of natural self-evidence (1971/2012, 26–35, 84–89; Summa, 2012, 2014). Broadly, these can be summarized according to (a) the *freedom*, namely in relation to the instantiation of the epoché as a change of attitude; (b) the *intensity*, namely in relation to the pervasiveness or radicality of the bracketing of the epoché.

In relation to (a) freedom, Blankenburg observes that the phenomenologist needs to stand against an “elastic force,” which Fink individuates as “*the tendency of life toward the world as its finality*” (Fink, 1988/1995, 24), to actually perform the epoché. In contrast, for individuals with schizophrenia, the epoché occurs *involuntarily*. As a result, the challenge for those affected by this condition is returning to the natural attitude, which characterizes our pre-reflective engagement with the everyday world. What is interesting in this regard is that, as Blankenburg observes, similarly but inversely to the phenomenologist, the person affected by schizophrenia experiences a kind of “resistance” (*Widerstandserfahrungen*), connected with the efforts of re-establishing a natural attitude through reflection.

In relation to (b) the intensity, while the phenomenologist through the epoché aims to suspend the so-called “general thesis” (*Generalthesis*), namely our passive beliefs concerning the natural world, (Husserl, 1913/1983, §27, §30–33) the schizophrenic epoché, on the contrary, also includes the totality of the pragmatic and affective sphere, deeply influencing the everyday life of the person affected by this condition. In this sense, the schizophrenic epoché is radical because it is not limited to our cognitive relation with reality, but it also encompasses our practical engagement. Therefore, because of these differences, Blankenburg acknowledges that the schizophrenic epoché cannot be ultimately reduced to the phenomenological epoché.

## 3. Situating Tatossian’s intrasubjective understanding of schizophrenia: the dualism of the transcendental life and the splitting of the ego

In his lecture “Délire: sujet et subjectivité” presented in Heidelberg in 1994, Tatossian criticizes the idea that schizophrenic alienation derives from a disorder of our intersubjective anchoring in the social and cultural world. Instead, he contends that schizophrenic autism originates from a “plus profonde” disturbance that can be found within the sphere of transcendental subjectivity. Namely, in the deeper structures of consciousness involved in the constitution of our experience and do not strictly depend on our relationship with others. Tatossian considers the atrophy of common sense and the loss of natural self-evidence characterizing schizophrenic existence as an effect or the “écho et la conséquence d’une disproportion antérieure et plus profonde de la *sphère intrasubjective*” (Tatossian, 2014, 288). In this sense, contrary to Blankenburg, he does not conceive schizophrenic autism as resulting from a loss of our originary sociality or primary intersubjectivity.

Since Tatossian conceptualizes schizophrenic existence by drawing on some ideas found in Fink’s *Sixth Cartesian Meditation*, it is essential to first clarify the conceptual presuppositions of his account before understanding this conception of schizophrenia. This is important, especially considering that Tatossian does not elaborate on his idea of schizophrenia by discussing a case study like Blankenburg. Indeed, although Tatossian does mention clinical examples, in the lecture he draws almost exclusively on theoretical considerations, so his account remains highly speculative. For these reasons, I further expand on his ideas to make sense of this intrasubjective account of schizophrenia.

Broadly, Blankenburg draws on the notion of phenomenological epoché, as it is presented by Husserl in *Ideas I*, to characterize schizophrenic alienation as a loss of natural self-evidence. Yet, by suspending our natural attitude, the phenomenological method does not only disclose the distinction between the transcendental, *constituting consciousness* and the *constituted world*. As Fink makes clear, on the one side, by bracketing all assumptions about the existence and reality of any object of experience, the phenomenologist finds himself as a *phenomenologizing onlooker*. In particular, the temporary suspension of the existence of any other subject compels the phenomenologist as an onlooker to adopt a somewhat “solipsistic” stance. Indeed, even if the “‘Others are transcendentally existent as constituting monads with whom the ego stands in a community of constitution,” this does not *phenomenologically* imply that they stand “in a community of transcendental self-knowledge” (Fink, 1988/1995, 123).

On the other side, by performing the intersubjective reduction, the phenomenologist acknowledges that, as constituting consciousness, it is essentially *communitarian* in its intentional performances. Indeed, by belonging to an ego-community, which includes individual ego-subjects, the phenomenologist recognizes the participation in a web of intersubjective meanings and practices that shape his or her experiences and understanding of the world. Therefore, while the egological reduction seems to emphasize the solipsistic character of the transcendental life, on the contrary, the intersubjective reduction seems to underscore its communitarian aspect. As Bernet writes, while “the constituting consciousness constitutes, orders and enriches the world, […] the spectator unfolds a phenomenological analysis of this uninterrupted work of constitution” (Bernet, 1994, 17). In this respect, while the phenomenologizing onlooker “acts in *solitude* […] different constituent subjects quite naturally associate and collaborate in shaping a world that is *common* to them” (Fink, 1988/1995, 17, my translation).

However, to provide more clarity on how the phenomenologist can detach himself from the world and, in doing so, adopt an objective perspective on their consciousness for intentional analyses, Husserl offers further insights in his lecture course *First Philosophy* from 1923/1924. For Husserl, our ability to distance ourselves from the natural attitude relies on a particular type of self-alienation, also known as Ego-splitting or doubling (*cf.* Cavallaro, 2020), that we experience in different kinds of acts. For instance, in natural self-reflection, we can direct our thematizing attention to some activity we perform by means of a “perception of a higher type” (Husserl, 2019, 291). Following Husserl’s example, in natural self-reflection, our perception of a house becomes the awareness of perceiving a house. In this respect, self-reflection is characterized by the simultaneous presence of two Egos, the Ego of perception and the Ego of reflection. Indeed, even though, at the onset of self-reflection, the “*emerging”* Ego of reflection takes the act of perceiving the house as its content in the form of the past, the positing of the reflecting Ego actually implies a “*coexistence* [of] *the doubled I and the doubled I-actus*” (*ibidem*, 292).

Arguably, in natural self-reflection, by participating in the interests of the lower I, the reflecting I is almost “identical to it in the *manner* of the interested position-taking, co-believing, co-assuming, co-doubting, and so on” (Husserl, 2019, 299–300). In contrast, as Husserl makes clear, since the phenomenologist needs to suspend the doxastic validity of the lower-level I to perform the phenomenological epoché, the simultaneous presence of two Is assumes a new meaning. Indeed, the renunciation of the “co-belief” between the two Is that characterizes our natural self-reflection suspends the very identity of the two Is. According to Husserl, this suspension is possible due to an originary freedom, which allows us to remain detached from the *existence* and the *manner* in which the perceived objects of perception manifest, even in relation to “the existence of the world as such” (*ibidem*, 295).

In this regard, Zahavi writes: “Becoming a theme to oneself is a matter of becoming divided from oneself. Reflective self-awareness involves a form of alienation. It is characterized by a type of self-fragmentation that we do not encounter on the level of pre-reflective self-awareness”^7^ (Zahavi, 2005, 91). Indeed, in performing the phenomenological epoché, the disinterestedness of the reflecting I cannot be reducible to a “*mere privation*, a mere passivity” (Husserl, 2019, 296). As Husserl argues, to become an “unparticipating observer” a “*special motivation*” is, in effect, necessary to “*free*” the phenomenologist from the “sympathy” of the interested Ego of perception. Hence, the phenomenologizing onlooker, as reflecting I, can practice the epoché because of the possibility to “distance” from the reality claims that belong to the lower-level I. In other words, it is only because it can be “alone,” in an originary sense, that the phenomenologist can suspend every natural position-taking.

In this respect, the phenomenological bracketing is a non-participating kind of self-reflection meant to become “active in a complete disinterestedness with respect to the intentional objectivity *as existing*” (Husserl, 2019, 311). It is precisely the possibility of *breaking the co-believing* of the two Egos, stacked upon each other, which allows the phenomenologist to reduce all experiences to transcendental consciousness.^8^ As I show in the following section, Tatossian characterizes schizophrenic alienation by reflecting on the implications of the loss of this ability, which, in a certain sense, constitutes the originary condition of the possibility of “being-alone” in the sense of being detached from one’s experience.^9^

### 3.1. Framing schizophrenic existence: on the dialectic between the solipsistic distancing from one’s experience and the communitarian constitution of the lifeworld

In his lecture “Délire: sujet et subjectivité,” Tatossian develops the idea that schizophrenic existence can be defined as a split between the twofold dimension of the transcendental life. Indeed, as he writes, in schizophrenic subjectivity “everything happens as if it merges in itself the characteristic attributes of the constituent consciousness and the ‘phenomenologizing spectator’ in the phenomenologist”^10^ (2014, 289). For Tatossian, the solipsistic character associated with the phenomenologizing onlooker, which, as Husserl makes clear, is the “high order” I of self-reflection – for its ability to distance from the world –, is taken up by the constituting consciousness. Tatossian justifies this claim by arguing that in full-blown schizophrenia there seems to be no trace of a self-reflecting I that can attribute to itself all his constitutive performances and meaning-bestowal achievements. In this respect, it is worth mentioning that, differently from Blankenburg’s seminal monography and contrary to his first positions (*cf.* Tatossian, 1979/2002, 2009), Tatossian seems to grant more attention to full-blown forms of schizophrenia to characterize the psychopathological transformations of lived experience that better define the basal disturbance of schizophrenia.

Indeed, Tatossian takes full-blown schizophrenia as an example of the breakdown of the coexistence of the two Is, or of the twofold dimension of the transcendental subjectivity, insofar as schizophrenic subjectivity finds itself alone in its own constituting “exubérance.” In this respect, in contrast to Blankenburg, who actually does not address how the loss of natural self-evidence relates to positive symptoms (*cf.* Sass, 2001, 261), Tatossian suggests that this split leads to the development of delusions and hallucinations because the individual constructs a psychopathological world that is disconnected from the shared lifeworld of the others. According to this reading, psychotic hallucinations, such as “hearing thoughts aloud,” must be read as the *perceptualization* and *spatialization* of inner speech, thoughts, and feelings. In this respect, Parnas and Sass, 2011 write that hyper-reflexivity, which characterizes schizophrenic experience, involves “a sense of *experiential fissure* between the sense of subjectivity and the contents of awareness. […] the thinking process seems to lose its sense of vital self-affection. Smooth, first-order thinking is undermined by a kind of ongoing introspection that imposes itself on the patient, but that may also be taken up by the patient as a more active project. As a result, mental contents appear increasingly objectified and spatialized (thinking may, e.g., be felt localized to specific parts of the head)” (Parnas and Sass, 2011, 535).

For this reason, differently from Blankenburg, Tatossian interprets schizophrenic alienation as a “plus qu’une pseudo-epoché,” or a “caricature” of the phenomenological epoché. Indeed, since the schizophrenic epoché lacks the modality of the spectator, namely the high-order self-reflecting I that attributes to himself the intentional performances that constitute his experience, it should not be possible to talk at all of a proper “reduction.” As Tatossian would suggest, the schizophrenic epoché would only present an *empty scene*. If, as he argues by drawing on Kimura Bin’s ideas, in full-blown schizophrenia there is a “noetic splitting” in the flow of consciousness, then there is more than one distinct subjectivity co-existing in a specific “*rapport d’influence*” (Tatossian, 2001, 67).^11^ This can be understood by theorizing that in schizophrenia the Ego-splitting or doubling as originary capacity of coming-to-ourself is profoundly impaired. In a healthy subjectivity, as Husserl observes, the whole “*egoic life in activity* is nothing but a *constantly-splitting-itself-in-active-comportment* and […] at all times anew an all-overlooking I can establish itself which identifies all [of those acts and act subjects]” (Husserl, 2019, 294). Instead, in schizophrenia what seems lacking or disturbed is, in this respect, the “synthetic identification of identity of sameness of all of these act poles and of the difference of their modal manners of existence” (*ibidem*, 294, 589–590).

Indeed, it is not by chance that Fink refers to the phenomenological reduction as a type of schizophrenia (*Art Schizophrenie*) (*cf.* Luft, 2002, 114). In this respect, Luft remarks that while the Ego-splitting allows the self-thematization of one’s own Ego because the Ego is self-objectivized in the act of self-reflection, there must be a synthesis of identification that unifies this duality. For Luft, this synthesis of identification is the synthesis of inner time consciousness that preserves the unity of the stream of consciousness (*Einheitlichkeit des Bewusstseinsstromes*), through a single continuous stream of living present experience (*Lebensstrom der lebendigen Gegenwart*). When this unity is disrupted, it is no longer possible to speak of a coherent self (*cf.* Tatossian, 2001, 67). In this respect, the temporal dimension of schizophrenic disturbances is very much at the center stage in contemporary research. Fuchs (2010) argues that disturbances of the constitutive synthesis of time consciousness can explain the fragmentation of loss of self-coherence and other self-disturbances. Fuchs considers the “*weakening and temporal fragmentation of self-experience* […] as a generative disturbance of schizophrenia” that would make the “loss of natural self-evidence” a secondary phenomenon resulting from a disturbance in the intentional arc (*cf.* 2010, 84). Similarly, Sass and Pienkos (2013, 141) argue that schizophrenia is characterized by a temporality that “loses all organization and meaning (*cf.* Fuchs, 2007; Vogeley and Kupke, 2007).

Tatossian further distinguishes the case of paranoid schizophrenia from non-severe forms. As he argues, in early schizophrenia an onlooker seems to be still present, in the form of a reflexive I that unifies all subjective acts. In this respect, he argues that what characterizes early schizophrenia is the “spectacle” of the world-constitution, that is, the ongoing process of producing the sense of the “world” through which “constituting subjectivity is at the same time constantly enworlded and constituted as humanity, as a totality of humans living with one another in open, finite mediation” (*cf.* Fink, 1988/1995, ft. 107). Against this background, Tatossian would explain the loss of common sense, which is the intersubjective product of world-constitution by the transcendental community of egos, as originating from the split between the self-reflecting I, which is, in this case, the I of self-awareness, with its constitutive achievements, in particular those that involve the others. Indeed, Tatossian seems to suggest that in pauci-symptomatic forms of schizophrenia the self becomes a pure “gaze,” “pure ipseity” (*ipséité*), which cannot appreciate his activity of constitution, “has nothing to look at, neither the world, nor the self” (Tatossian, 2001, 67, my translation). This loss of the world-constituting capabilities of the transcendental consciousness actually echoes Blankenburg’s intuition concerning the atrophy of Anne Rau’s “world-forming force” (*weltbildende Kraft*) that is responsible for the constitution of experience, resulting in a loss of spontaneity or naturality that she finds in the others.

By drawing again on the analyses of Kimura (1992), Tatossian interprets the non-delirious schizophrenic as someone who cannot form any “objective noematic self” (*soi objectif noématique*). Instead, schizophrenic subjectivity is stuck as a noetic self in two simultaneous moments of seeing and being seen. Tatossian defines this type of self-alteration as the experience of being other than oneself. In this respect, Nagai has described this experience as the experience of “simultaneous introspection,” namely “two *Is*” that simultaneously observe each other (Motobayashi et al., 2016, 497). She considers the excess of introspection to be a fundamental phenomenon in schizophrenia. Similarly, and more recently, Stephensen and Parnas write that in schizophrenia there is a kind of “involuntary self-witnessing,” which entails a loss of the unity of experience. This implies that individuals with schizophrenia experience a “fundamental self-fragmentation or duplication” within their own stream of consciousness (2018, 635). This split or duplication is also associated with the loss of a “clear-cut subject-object structure” (*cf.* Henriksen et al., 2019, 7). In this respect, Stephensen and Parnas argue that the “pronounced self-duplication or self-redoubling in schizophrenic self-experience is a consequence of a vulnerability in the functioning auto-affection, which normally assures the feeling of self-coincidence in the constant differentiation and reintegration of the subject” (2018, 639).

To conclude this brief discussion on Tatossian’s idea of schizophrenic alienation, it is worth stressing that, for Tatossian the split between the two Is, which are understood within the phenomenological theory of method as the phenomenological onlooker and the constituting consciousness, does not depend on some disturbances of our intersubjective anchoring in the lifeworld. Yet, Tatossian does not delve into the crucial problem of whether our ability to distance ourselves from the world and to observe our experience is actually independent of our interactions with others or whether it is the product of intersubjective synthesis. In this regard, the difference between an intersubjective and an intrasubjective account of schizophrenia amounts to the problem of whether the splitting or the duplication can be considered primary or it is rather “a form of ‘phenomenological compensation’” (Stephensen and Parnas, 2018, 639; Sass, 2001, 265–266). In this respect, as I have shown, whereas Blankenburg considers the disturbances of reflectivity as compensatory compared to the disturbance in the pre-reflective attunement to the world as a “loss of natural self-evidence” (*cf.* Sass, 2001), Tatossian seems to take the disturbances of self-reflective awareness as primary.

## 4. Conclusion

In this paper, I have presented two distinct perspectives on schizophrenia. I have shown that the difference between Blankenburg’s and Tatossian’s interpretations of schizophrenia stems from their respective phenomenological assumptions. For Blankenburg, the basal disturbance of schizophrenia is a transformation of the anchoring (*Verankerung*) of the human being into the lifeworld. Blankenburg’s attention on the intersubjective sphere to frame schizophrenic disturbances is already evident in the article of 1969, where he rejects the idea that schizophrenia could be understood as a disturbance of cognition or affectivity and writes that “Affectivity and the ability to judge, as we find it in common sense, refer back to an original unity of thinking, feeling, and willing in human existence, which is primarily related to an intersubjective world (*mitweltbezogen*)” (1969/2001, 307).

Blankenburg conceives schizophrenia as a psychopathology of common sense by placing emphasis on impairments in the intersubjective dimension of experience (*cf.* Mishara, 2001; Summa, 2012, 2014). Indeed, for him, “Seen in terms of transcendental phenomenology, the peculiar ability of common sense has its basis in the intersubjective constitution of the life-world” (Blankenburg, 1969/2001, 310). In this model, schizophrenic autism derives from a loss of natural self-evidence, which refers to our typical understanding of the practical and social world. This primary phenomenon consequently leads to alienation from the world of social interactions (secondary autism) (Blankenburg, 1969/2001, 1986; Pienkos, 2015; Sass and Pienkos, 2015). Indeed, it is not by chance that the analysis of Anne’s intersubjectivity is the lengthiest of his monography. In this respect, Blankenburg draws on the later Husserlian works on genetic and generative phenomenology, even though he does not elaborate on a genetic understanding of Anne Rau’s schizophrenic experience.

By considering the impairment of the second- and first-person plural awareness as the most important alterations of lived experience characterizing early schizophrenia (*cf.* Stanghellini and Lysaker, 2007), Blankenburg actually broadens the limits of psychopathological investigation. In this sense, as Thoma et al. (2022) have recently stressed, Blankenburg’s late works call “for an analysis not only of the self but even more of the social and cultural context, i.e., the different *lifeworlds* in which the constitution of everydayness is inhibited” (2022, 5). Indeed, since he became increasingly interested in understanding how the familial context may constitute a risk factor in the development of schizophrenia (Blankenburg et al., 1983), Blankenburg has *de facto* expanded the classical phenomenological accounts of schizophrenia by including sociological and etno-psychiatric considerations. As Thoma et al. (2022) show, Blankenburg’s later works consider schizophrenia as an “attempted solution to a problem in social structures, more specifically to the problem and challenge of emancipation from a specific family milieu” (2022, 10; *cf.* Blankenburg, 2007). However, in his account, Blankenburg leaves open the problem of the relationship between the factual becoming and the genetic constitution (*faktischem Werden und konstitutiver Genesis*) of Anne Rau’s experience. In this regard, at the end of section IX of his monography, Blankenburg seems to acknowledge the lack of antepredicative analyses in genetic phenomenology (Blankenburg, 1971/2012, 150, 170–179).^12^

Tatossian, on the contrary, grounding his intrasubjective reading of the basal disturbance of schizophrenia on Fink’s idea of a duality of the transcendental life, interprets schizophrenic subjectivity in the sense of a split between two Is normally coexistent in a healthy subjectivity. To be clear, on the basis of a reading of the “transcendental theory of reduction,” Tatossian develops a comparison between the fragmented nature of schizophrenic subjectivity and the split between the constituting consciousness and the phenomenologizing onlooker. I have further developed Tatossian’s by relying on the idea of Ego-splitting, which can be found in Husserl’s phenomenological-psychological theory of epoché and reduction. Typically, the self-alteration produced by the splitting or doubling of our experience is non-pathologic, since it is a necessary condition for our acts of reflection, representation, and imagination (*cf.* Cavallaro, 2020). However, the splitting assumes a new meaning in schizophrenia because self-duplication loses its dynamic character and becomes a pathological type of self-fragmentation. As Sass (2007), self-fragmentation encompasses thoughts, perception, and actions, with “popping-out” experiences, such as emerging inner speech or kinesthetic sensations, and a general disorganization and weakening of the field of awareness (*cf.* Sass, 2018b, 601).

In this sense, I have shown that for Tatossian schizophrenic autism primarily derives from a disturbance of self-awareness, and, in particular, from a type of self-division that leads to a pathological form of self-alienation. Indeed, in his account, Tatossian draws on Nagai (Motobayashi et al., 2016) and Kimura’s (1992) analyses of the split between the noetic and noematic self or individual and collective subjectivity in schizophrenia. From this perspective, Tatossian’s interpretation is close to the *ipseity-disturbance* model. Sass and Parnas (2007, 63) argue that the basal disturbance of schizophrenic experience can be characterized by (a) hyper-reflexivity, which designates second-order mental states such as self-monitoring (*cf.* Sass, 2018a), and (b) diminished self-affection, which characterizes a decreased self-awareness and sense of agency. According to this account, schizophrenia involves a disorder of the minimal self, that is, our pre-reflective first-person presence that characterizes our experiential life in general and constitutes the *mineness* of our experience (*cf.* Parnas, 2000; Sass and Parnas, 2003; Zahavi, 2005; Henriksen et al., 2019). In particular, Henriksen et al. (2019) argue that what is lacking or transformed in schizophrenia is the “warmth and intimacy” of the lived experience. However, even if Tatossian seems to believe in a sort of ontological priority of the intrasubjective transformations of subjectivity in schizophrenia, he does not provide any genetic explanation of the reason why, for instance, our ability to distance from the world does not strictly depend on the constitution of the others or on we-syntheses.

As I have shown, the difference in their interpretation of schizophrenic disproportion can be attributed to their reading of the meaning of phenomenological epoché and reduction. Not because they purely apply the epoché and reduction, but because they rely on these notions to actually understand the functioning of schizophrenic subjectivity. Blankenburg uses the phenomenological epoché to conceptualize, by means of some important distinctions, schizophrenic alienation as a loss - and not simply a bracketing - of natural self-evidence, which is, ultimately, intersubjectivity constituted in our everyday engagement within the lifeworld. In contrast, in Tatossian’s account, the phenomenological epoché is used to characterize the transformations of schizophrenic alienation as a modification of the relationship between the phenomenologizing onlooker and the constituting consciousness.

While Blankenburg and Tatossian seem to present two apparently opposite accounts of schizophrenia, actually there are some fundamental similarities. Tatossian’s approach to schizophrenia is dialectic like that of Blankenburg (1982, 2007) but it differs for the object of this dialectic. Blankenburg conceives schizophrenia as deriving from a disturbance in the balance between what we naturally take as self-evident and what is problematic or non-evident. Indeed, it is an open question establishing to what extent the loss of natural evidence becomes *de facto* pathological. Instead, Tatossian proposes to consider schizophrenia as a disturbance in the balance between our ability to distance ourselves from the world and from our own position-takings and our ability to immerse ourselves in the world with others and to actively engage with it. In this sense, he suggests that schizophrenic existence is characterized by a deficit of self-intimacy because, from this perspective, it is “the incapability to be alone which best characterizes and founds the incapability to meet Others”^13^ (2014, 290, my translation).

In my account, these perspectives are mutually complementary in a methodological sense because they reflect on the psychopathological disturbances by considering two co-originary poles of Husserl’s transcendental phenomenology as *a priori* conditions of experience in general, altogether with the world (*cf.* Zahavi, 2003; Taipale, 2014; Zhang, 2021). Indeed, as Husserl acknowledges, our intentional relation to the world already implies an implicit “reference to other subjects prior to any concrete experience of them” because “my perceptual intentionality contains a reference to Others, regardless of whether or not I experience these Others, and, indeed, regardless of whether they actually exist or not” (*cf.* Zahavi, 2003, 118–119). As Husserl also argues, if “one interprets transcendental subjectivity as an isolated ego and in the spirit of the Kantian tradition ignores the whole task of establishing a transcendental community of subjects, then every chance of reaching a transcendental self- and world-knowledge is lost” (*cf.* Zahavi, 1996, 1; Husserl, 1993, 120). In this sense, these two perspectives on schizophrenia are mutually enriching since they shed light on different but interrelated disturbances of experience. Arguably, before solving the problem of the explanatory relationship between levels or dimensions of psychopathological alterations of lived experience (*cf.* Sass, 2014), a pluralistic approach to the basal disturbances of schizophrenia is epistemologically justified because it does not favor particular ontological presuppositions to avoid oversimplifications (*cf.* Sass et al., 2018, 722).

Indeed, the debate on the explanatory relationship between different psychopathological phenomena in schizophrenia is still open. Actually, one of the main problems in the philosophy of psychiatry is understanding the relevance and contribution of each level of explanation to the conceptualization of psychiatric illness (*cf.* Kendler, 2005; Murphy, 2008). This is due to the inherent complexity of “distinguishing between more fundamental, enduring, trait-like features that are largely automatic or passively experienced […] and more consequential or compensatory features that develop largely in response” (Sass et al., 2018, 722).^14^ Similarly, in transcendental phenomenology, as Zahavi, 2017, writes, “to argue that self, world, and others are all involved in the constitutive process is not to say that they are all involved in the same way, or all involved on all levels” (Zahavi, 2017, 128). In this regard, because of the lack of a proper explanation of the “zone d’insertion du processus psychotique” in schizophrenia (Tatossian, 2014, 288), I have avoided a one-sided view of schizophrenia. Instead, I have taken a pluralistic approach in examining two classical accounts of schizophrenic existence, with the aim of investigating how classical phenomenological concepts can contribute to our understanding of psychopathological conditions.

## Author contributions

The author confirms being the sole contributor of this work and has approved it for publication.

## Funding

The research conducted in this publication was funded by the Irish Research Council under grant number “GOIPG/2021/606.”

## Conflict of interest

The author declares that the research was conducted in the absence of any commercial or financial relationships that could be construed as a potential conflict of interest.

## Publisher’s note

All claims expressed in this article are solely those of the authors and do not necessarily represent those of their affiliated organizations, or those of the publisher, the editors and the reviewers. Any product that may be evaluated in this article, or claim that may be made by its manufacturer, is not guaranteed or endorsed by the publisher.

## Footnotes

^1^The idea of a “basal disturbance” is associated with essentialist accounts of mental disorders that propose a unifying essence or concept to capture various symptoms of a psychopathological condition (*cf.* Sass, 2010). In this respect, while Blankenburg denies that his essentialist characterization of schizophrenia can have an etiological relevance, he acknowledges the explanatory power of such approach for grasping the typical manifestations of this disorder (1971/2012, 4, 27).

^2^In this respect, Zahavi (2021) warns against the risk of using the epoché and reduction for “non-philosophical application of phenomenology” (2021, 262). As he argues, Husserl elaborated these phenomenological ideas in the context of his *transcendental idealism*, to make intelligible “the universal obviousness of the being of the world” (*ibidem*, 262; Husserl, 1936/1970, 180). For him, the epoché and reduction may mislead qualitative researchers by overwhelming them with “methodological metareflections” (Zahavi, 2021, 268). As he argues, studying psychic states does not necessarily require such methods, as our natural engagement with the world already involves understanding others through a personalistic attitude. Also, the epoché and reduction are not the sole means of adopting a critical stance toward metaphysical and epistemological assumptions. The focus on the epoché and reduction may hide the fact that phenomenology offers “theoretical accounts of its own” (*ibidem*, 271).

^3^With this expression, Blankenburg refers to the structure of the conditions of possibility of the operative relation to the self and the world in a given human *Dasein*. In this respect, Blankenburg substitutes “constitution of the Dasein” with “transcendental organization” to avoid the static character of Binswanger’s expression and to consider the *a priori* structures of existence from a genetic perspective (*cf.* Blankenburg, 1962). However, Blankenburg integrates the *daseinanalytic* concept of “being-in-the-world” with the phenomenological idea of a “transcendental organization” of experience. Indeed, his provisional abandonment of the *daseinanalytic* problems of biography is a strategical, for it allows him to focus exclusively on the pre-predicative level of experience and in general on constitutive problems.

^4^Notably, in the postface of the French edition, Blankenburg admits the nosological difficulties concerning Anna Rau’s diagnosis, whether it was a case of schizophrenia simplex or borderline syndrome. Yet, he replies that even though this diagnostic problem is justified, his work was not “about nosological questions in the service of which phenomenological analyses would have been conducted, as in Jaspers, K. Schneider, G. Huber and others. Schneider, G. Huber among others, but of an access to the view of the phenomena of the psychic life as phenomena” (Blankenburg, 1971/1991, 232–233, my translation).

^5^“Ich finde einfach, daß ich noch den Halt brauche. Bei den allereinfachsten alltäglichen Sachen brauche ich Halt. […] Das ist wohl die natürliche Selbstverständlichkeit, die mir fehlt” […] Da ist mir vieles fremd. Wenn die anderen so handeln, und jeder ist eigentlich so irgendwie groß geworden: Danach denkt man, danach ist das Handeln ausgerichtet, danach verhält man sich. (…) Und auch ebenso die Denkarten, so das Einfache, das Einfachste. Jeder Mensch ist doch etwas. Jeder bewegt sich doch in einer Bahn. Das ist eben bei mir nicht der Fall. Einfach um das Leben geht es, um ein richtiges Leben-Führen, dass man nicht so außerhalb – so außerhalb der Gesellschaft, so ausgestoßen ist und so” (*cf.* also Summa, 2012, 193).

^6^It is worth stressing that, for Blankenburg, the eidetic reduction does not need to be preceded by a phenomenological epoché but can be independent, as he argues in the text from 1980. This is because Blankenburg relies on Goethe’s metamorphosis theory of the aboriginal plant (“*Urpflanze*”), according to which it is possible to focus on the *Gestalt* of the phenomena for “tracing the similarities of different - in their fully developed state apparently disparate (as healthy and pathological) - regions of phenomena” (1980, 57).

^7^Zahavi observes that reflective self-awareness is distinguished from pre-reflective awareness “by a certain detachment and withdrawal, since reflection deprives the original experience of its naïveté and spontaneity. To put it another way, reflective self-awareness does not differ from pre-reflective self-awareness merely by its intensity, articulation, and differentiation, but also by its quality of othering” (2005, 91). In this respect, Zahavi argues that reflective self-awareness is grounded on “the unity of unification and differentiation inherent in the pre-reflective lived presence: its ecstatic-centered structure of protending, presencing, retaining.” (*ibidem*, 92) Therefore, self-alteration is already a possibility inscribed in the very structure of inner time consciousness, which designates the dynamic, temporal self-differentiation.

^8^As Fink writes: “In the performance of the phenomenological reduction, transcendental life, in producing the “onlooker,” ‘steps *outside itself*, splits itself, divides. This dividing [*Entzweiung*], however, is the *condition of the possibility of coming­to­itself* for transcendental subjectivity” (1988/1995, 23).

^9^In this regard, Moran, (2001) discussing reflective distancing writes that: “Stepping back” from an impulse of mine is a metaphor of distancing, obviously; but also one of observation and bringing into view, and of separation and distinguishing (as in the sense that this impulse is one thing and I another), and of confrontation and facing (suggesting something unavoidably in my path). […] “stepping back” involves the halting of one’s forward movement, the interruption of an action, suspending one’s motion in pursuit of some aim” (2001, 143).

^10^“[…] tout se passant comme si elle fusionnait en elle-même les attributs caractéristiques de la conscience constituante et du ‘spectateur phénoménologisant’ chez le phénoménologue.”

^11^With “noetic self,” Kimura intends the auto-perception or self-awakening (*jikaku*) of the self as mental process, while for “noematic self” he intends a representation or an objectification of the noetic self *cf.* Pélicier (1984, 32). For Kimura the basal disturbance of schizophrenia must be searched in a “kind of intrapsychic splitting” between “the I that is represented in human self-consciousness and the subject itself as the vital ground of this self-consciousness” (Kimura, 2001, 335). Kimura also talks of a “discordance” between the dual nature of subjectivity, as individual and collective at once. Whereas individual subjectivity is characterized by a sense of inner I-ness, the “the I-ness of the self or the selfness of the I,” the collective subjectivity designates the individual in group (2001, 336).

^12^“Wir wissen gegenwärtig noch viel zu wenig über die Eigenarten des vorprädikativen Weltbezugs und über seine Variabilität.”

^13^“l’incapacité à être seul qui le caractérise le mieux et fonde l’incapacité à rencontrer Autrui.”

^14^Yet, this difficulty does not limit Sass and Parnas (2003) to taking diminished self-affection and hyperreflectivity as equiprimordial alterations of awareness in schizophrenia (*cf.* Sass and Parnas, 2007; Sass, 2010, 2014). However, in this respect, de Haan and Fuchs (2010) consider these phenomena as a “compensatory strategy ensuing from the disturbances at the basic, embodied level.” because, in their model, schizophrenia is understood as a disturbance of the bodily self (2010, fn. 332).
